# Differential Regulations of Antioxidant Metabolism and Cold-Responsive Genes in Three Bermudagrass Genotypes under Chilling and Freezing Stress

**DOI:** 10.3390/ijms241814070

**Published:** 2023-09-14

**Authors:** Zhou Li, Cheng Huang, Liebao Han

**Affiliations:** 1 Institute of Turfgrass Science, Beijing Forestry University, Beijing 100083, China; 2 Department of Turf Science and Engineering, Sichuan Agricultural University, Chengdu 611130, China

**Keywords:** ascorbic acid–glutathione cycle, ethylene response pathway, gene expression, heat shock factor, heat shock protein, osmotin

## Abstract

As a typical warm-season grass, bermudagrass growth and turf quality begin to decrease when the environmental temperature drops below 20 °C. The current study investigated the differential responses of three bermudagrass genotypes to chilling stress (8/4 °C) for 15 days and then freezing stress (2/−2 °C) for 2 days. The three genotypes exhibited significant variation in chilling and freezing tolerance, and Chuannong-3, common bermudagrass 001, and Tifdwarf were ranked as cold-tolerant, -intermediate, and -sensitive genotypes based on evaluations of chlorophyll content, the photochemical efficiency of photosystem II, oxidative damage, and cell membrane stability, respectively. Chuannong-3 achieved better tolerance through enhancing the antioxidant defense system to stabilize cell membrane and reactive oxygen species homeostasis after being subjected to chilling and freezing stresses. Chuannong-3 also downregulated the ethylene signaling pathway by improving *CdCTR1* expression and suppressing the transcript levels of *CdEIN3-1* and *CdEIN3-2*; however, it upregulated the hydrogen sulfide signaling pathway via an increase in *CdISCS* expression under cold stress. In addition, the molecular basis of cold tolerance could be associated with the mediation of key genes in the heat shock pathway (*CdHSFA-2b*, *CdHSBP-1*, *CdHSP22*, and *CdHSP40*) and the *CdOSMOTIN* in Chuannong-3 because the accumulation of stress-defensive proteins, including heat shock proteins and osmotin, plays a positive role in osmoprotection, osmotic adjustment, or the repair of denatured proteins as molecular chaperones under cold stress. The current findings give an insight into the physiological and molecular mechanisms of cold tolerance in the new cultivar Chuannong-3, which provides valuable information for turfgrass breeders and practitioners.

## 1. Introduction

The bermudagrass (*Cynodon* spp.) species is frequently used for landscaping, athletic fields, and the conservation of water and soil in temperate regions or transition zones due to its many economical characteristics such as fast regrowth, fine turf quality, and strong tolerance to high temperature and drought stress [1,2,3]. As a typical carbon four (C4) plant, bermudagrass is susceptible to cold stress which has become a major environmental constraint for its utilization worldwide [4]. Optimal temperature ranges for bermudagrass growth and development are between 26 and 35 °C. Chilling stress (CS) is a problem, as an environmental temperature of 0–15 °C stops bermudagrass growth and also accelerates leaf chlorosis and senescence. When the ambient temperature is below 0 °C, freezing stress (FS) induces cell dehydration and the quick formation of ice crystals in plant cells [5,6,7]. It has been documented that an enhanced antioxidant defense system protects bermudagrass from cold-induced oxidative damage since cold stress triggers high levels of reactive oxygen species (ROS) production [8]. Superoxide dismutase (SOD) is regarded as the first line of antioxidant defense against ROS because it catalyzes the dismutation of superoxide anion (O_2_^−^) to form hydrogen peroxide (H_2_O_2_). Catalase (CAT) and peroxidase (POD) are two H_2_O_2_-scavenging enzymes under cold stress [9]. Enzymes including ascorbate peroxidase (APX), monodehydroascorbate reductase (MDHAR), dehydroascorbate reductase (DHAR), glutathione reductase (GR), glutathione S-transferase (GST), glutathione peroxidase (GPX), as well as non-enzymatic antioxidant metabolites such as reduced ascorbate (ASA), dehydroascorbic acid (DHA), reduced glutathione (GSH), and oxidized glutathione (GSSG) in the ASA-GSH cycle, also play vital roles in H_2_O_2_ detoxification in plants under various abiotic stresses [10,11]. Different plant species may vary largely in regulating antioxidant pathways to adapt to CS and FS. For example, the chilling-tolerant pangola grass (*Digitaria eriantha*) cultivar Mejorada INTA accumulated more GSH rather than regulating SOD and APX activities to alleviate CS-induced oxidative damage [12]. However, as compared to the chilling-sensitive chickpea (*Cicer arietinum*) genotype ILC533, the cold-tolerant genotype Sel96th11439 exhibited lower oxidative damage through enhancing SOD and CAT activities along with the unchanged activities of POD and APX in response to CS [13].

In addition to the antioxidant defense system, plants develop various adaptive mechanisms for better survival under chilling and freezing conditions [14,15,16]. The c-repeat binding factor (CBF) pathway is one of the most important defensive mechanisms against CS and FS in plants [17,18]. The ethylene (ETH) signaling pathway mediates plant growth and development, senescence, and stress adaptation [19,20]. An enhanced ETH pathway significantly decreased the freezing tolerance of *Arabidopsis thaliana* associated with inhibited *CBFs’* expression, whereas an ETH biosynthesis inhibitor could increase the freezing tolerance of *Arabidopsis thaliana* plants, indicating a negative role of ETH in regulating FS response in plants [21]. The exogenous application of an ETH precursor could also confer increased sensitivity to CS in wild-type bermudagrass [22]. Hydrogen sulfide (H_2_S) is another gaseous signal molecule that plays a positive role in the mediation of plant responses to CS and FS [23]. It was demonstrated that CS triggered H_2_S signaling in *Vitis vinifera* seedlings, and their chilling tolerance was increased, which was associated with enhancements in *VvCBF3* expression and antioxidant capacity to relieve toxic ROS when the seedlings were pretreated with an H_2_S donor [24]. Similar results were found in the study of Shi et al., who demonstrated that H_2_S donors improved the ASA–GSH cycle, contributing to ROS homeostasis under CS, but H_2_S scavenging exhibited an adverse effect on the chilling tolerance of *Arabidopsis thaliana* [25]. It is noteworthy that the accumulation of stress-induced proteins, such as heat shock proteins (HSPs) and osmotin, is another important strategy for adapting to cold stress in plants because of the beneficial roles of these protective proteins in osmoprotection, osmotic adjustment, and the repair of denatured proteins as molecular chaperones under CS and FS conditions [26,27]. 

Management strategies such as the use of protective winter covers and late-season fertilization have been reported to reduce cold injury to bermudagrass [28,29]. However, new cold-tolerant variety selections are often propitious to decreasing maintenance and management costs. In 2020, a new hybrid bermudagrass (*C. dactylon* × *C. transvaalensis*) cultivar Chuannong-3 with delayed leaf yellowing in winter and rapid spring green-up was licensed in China. Sugar accumulation and metabolism, the abscisic acid (ABA)-regulated pathway, and the CBF1 pathway contributed to the superior cold tolerance of Chuannong-3 as compared to common bermudagrass (*C. dactylon*) 001 and hybrid Tifdwarf which are used as a commercial cultivar for establishing sports turf worldwide [30]. As mentioned above, various defensive responses are involved in cold adaptation in plants and the underlying mechanism of cold tolerance in Chuannong-3 remains to be further elucidated. The current study investigated whether the better cold tolerance of Chuannong-3 could be related to enzymatic and non-enzymatic antioxidant metabolism, heat shock and ETH signaling pathways, and key genes encoding osmotin and cysteine desulfurase for H_2_S signaling. A deep understanding of metabolic and molecular bases of chilling and freezing tolerances in Chuannong-3 is important for future bermudagrass breeding.

## 2. Results

### 2.1. Chlorophyll Content and Photochemical Efficiency in Response to Chilling and Freezing Stress

**Figure 1:**
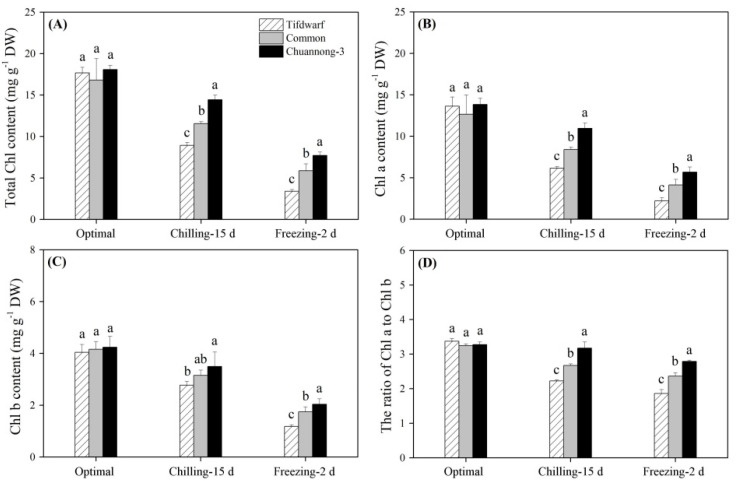
Changes in (**A**) total Chl content, (**B**) Chl a content, (**C**) Chl b content (OP), and (**D**) the ratio of Chl a to Chl b among three bermudagrass genotypes (Tifdwarf, common bermudagrass, and Chuannong-3) under optimal, chilling, and freezing conditions. Vertical bars indicate positive standard error of mean (*n* = 4). Different letters above columns indicate significant differences under optimal, chilling, or freezing condition based on least significant difference (*p* < 0.05), respectively.

**Figure 2:**
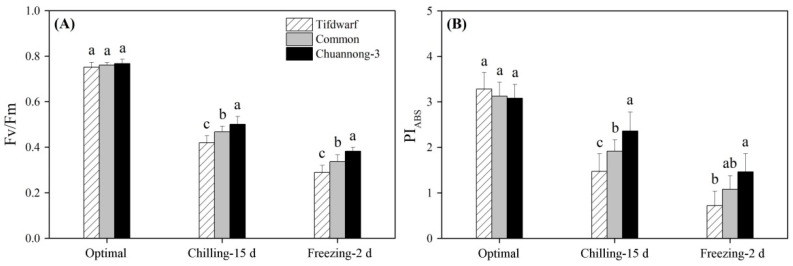
Changes in (**A**) Fv/Fm, and (**B**) PI_ABS_ among three bermudagrass genotypes (Tifdwarf, common bermudagrass, and Chuannong-3) under optimal, chilling, and freezing conditions. Vertical bars indicate positive standard error of mean (*n* = 4). Different letters above columns indicate significant differences under optimal, chilling, or freezing condition based on least significant difference (*p* < 0.05), respectively.

Total chlorophyll (Chl), Chl a, and Chl b contents as well as the ratio of Chl a to Chl b were not significantly different among the three genotypes under optimal conditions (Figure 1A–D). CS and FS induced significant declines in total Chl, Chl a, and Chl b contents, but Chuannong-3 maintained the highest total Chl, Chl a, and Chl b contents compared to Tifdwarf and common bermudagrass under CS and FS (Figure 1A–C). A similar trend was observed in the ratio of Chl a to Chl b among the three genotypes in response to CS and FS (Figure 1D). CS and FS significantly inhibited photosystem II photochemical efficiency (Fv/Fm) and performance index on absorption basis (PI_ABS_) in all plants (Figure 2A,B). Tifdwarf and Chuannong-3 exhibited the lowest and the highest Fv/Fm compared to the other genotype under CS and FS, respectively (Figure 2A). Chuannong-3 had 1.6 and 2.0 times higher PI_ABS_ than Tifdwarf under CS and FS, respectively (Figure 2B).

### 2.2. Antioxidant Metabolism in Response to Chilling and Freezing Stress

**Figure 3:**
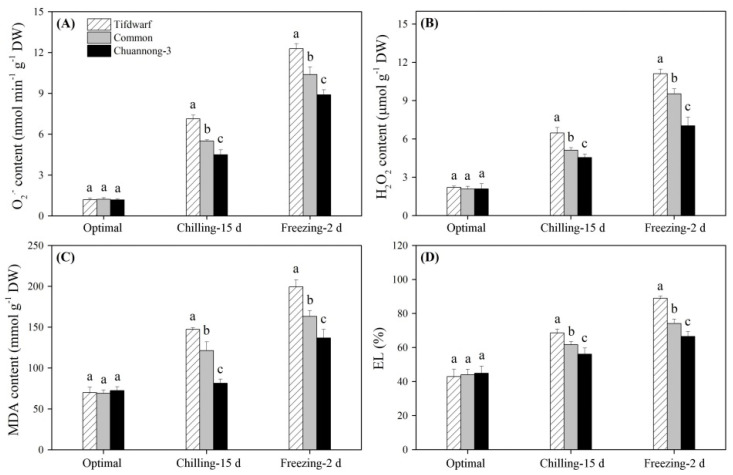
Changes in (**A**) O_2_^−^ content, (**B**) H_2_O_2_ content, (**C**) MDA content, and (**D**) EL among three bermudagrass genotypes (Tifdwarf, common bermudagrass, and Chuannong-3) under optimal, chilling, and freezing conditions. Vertical bars indicate positive standard error of mean (*n* = 4). Different letters above columns indicate significant differences under optimal, chilling, or freezing condition based on least significant difference (*p* < 0.05), respectively.

**Figure 4:**
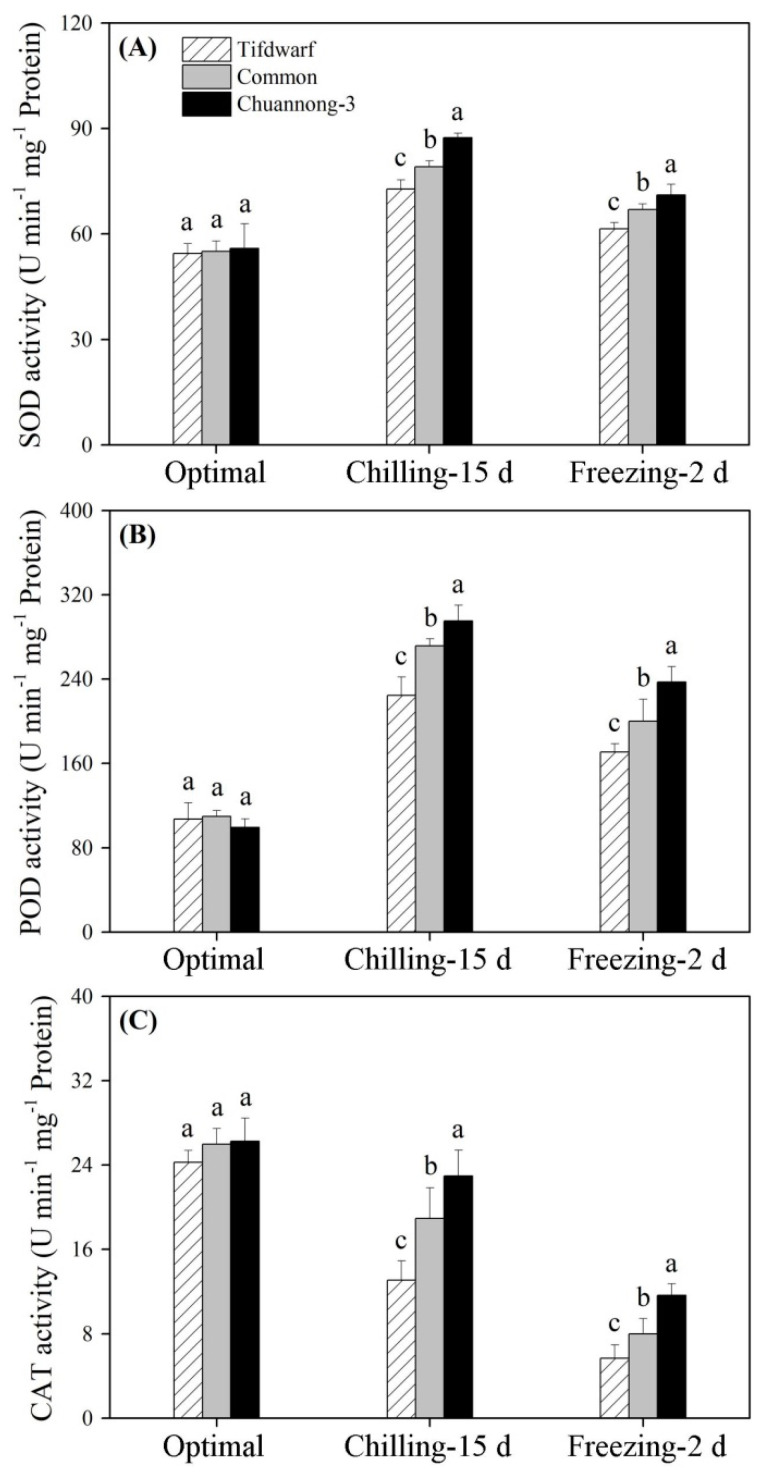
Changes in (**A**) SOD activity, (**B**) POD activity, and (**C**) CAT activity among three bermudagrass genotypes (Tifdwarf, common bermudagrass, and Chuannong-3) under optimal, chilling, and freezing conditions. Vertical bars indicate positive standard error of mean (*n* = 4). Different letters above columns indicate significant differences under optimal, chilling, or freezing condition based on least significant difference (*p* < 0.05), respectively.

The accumulation of O_2_^−^, H_2_O_2_, and malondialdehyde (MDA) was significantly induced by CS and FS in the three genotypes (Figure 3A–C). Electrolyte leakage (EL) also increased significantly when the three genotypes were subjected to CS and FS (Figure 3D). Chuannong-3, common bermudagrass, and Tifdwarf exhibited the lowest, the second highest, and the highest accumulations of O_2_^−^, H_2_O_2_, and MDA, as well as EL level, under FS, respectively (Figure 3A–D). CS activated SOD and POD activities, but decreased CAT activity in the three genotypes (Figure 4A–C). In response to FS, Chuannong-3 showed significantly higher SOD, POD, and CAT activities than Tifdwarf and common bermudagrass, respectively (Figure 4A–C).

**Figure 5:**
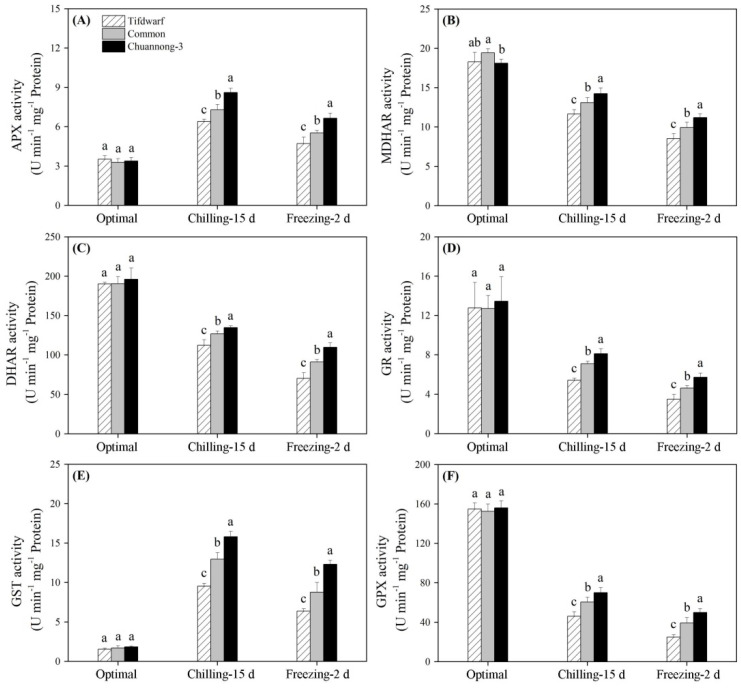
Changes in (**A**) APX activity, (**B**) MDHAR activity, (**C**) DHAR activity, (**D**) GR activity, (**E**) GST, and (**F**) GPX among three bermudagrass genotypes (Tifdwarf, common bermudagrass, and Chuannong-3) under optimal, chilling, and freezing conditions. Vertical bars indicate positive standard error of mean (*n* = 4). Different letters above columns indicate significant differences under optimal, chilling, or freezing condition based on least significant difference (*p* < 0.05), respectively.

**Figure 6:**
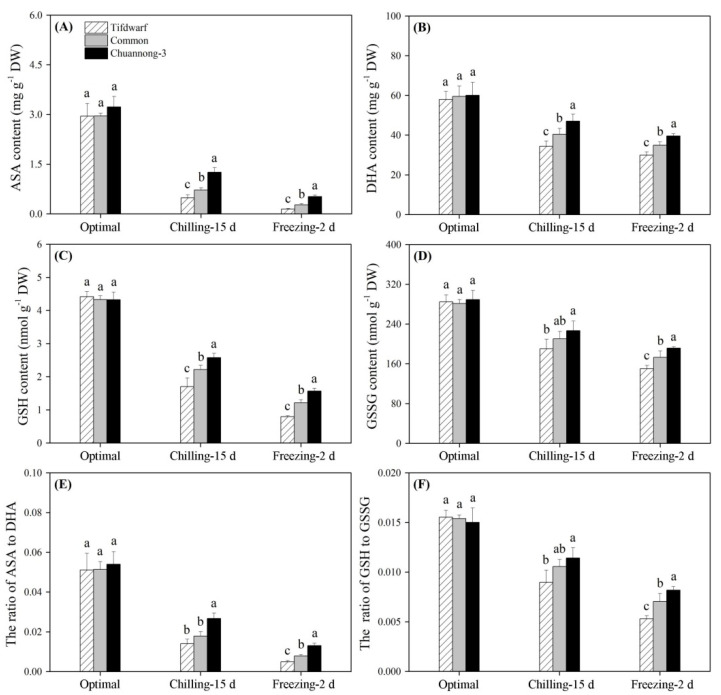
Changes in (**A**) ASA content, (**B**) DHA content, (**C**) GSH content, (**D**) GSSG, (**E**) The ratio of ASA to DHA, and (**F**) the ratio of GSH to GSSG among three bermudagrass genotypes (Tifdwarf, common bermudagrass, and Chuannong-3) under optimal, chilling, and freezing conditions. Vertical bars indicate positive standard error of mean (*n* = 4). Different letters above columns indicate significant differences under optimal, chilling, or freezing condition based on least significant difference (*p* < 0.05), respectively.

For enzyme activities involved in the ASA–GSH cycle, CS and FS up-regulated APX and GST activities, but down-regulated MDHAR, DHAR, GR, and GPX activities in all the genotypes (Figure 5A–F). The highest APX, MDHAR, DHAR, GR, GST, and GPX activities were detected in the Chuannong-3 compared to the other genotypes under CS and FS (Figure 5A–F). CS and FS not only inhibited accumulations of ASA, DHA, GSH, and GSSG in all genotypes, but also decreased the ratio of ASA to DHA, and GSH to GSSG (Figure 6A–F). Chuannong-3 had 1.9 and 3.5 times higher ASA content than common bermudagrass and Tifdwarf under FS, respectively (Figure 6A). The lowest GSH content was observed in the Tifdwarf as compared to the other genotypes under FS (Figure 6C). Chuannong-3 maintained the highest the ratio of ASA to DHA and the highest ratio of GSH to GSSG than the other genotypes under CS and FS (Figure 6E,F).

### 2.3. Differential Expression of Cold-Responsive Genes in Three Bermudagrass Genotypes under Chilling and Freezing Stress

**Figure 7:**
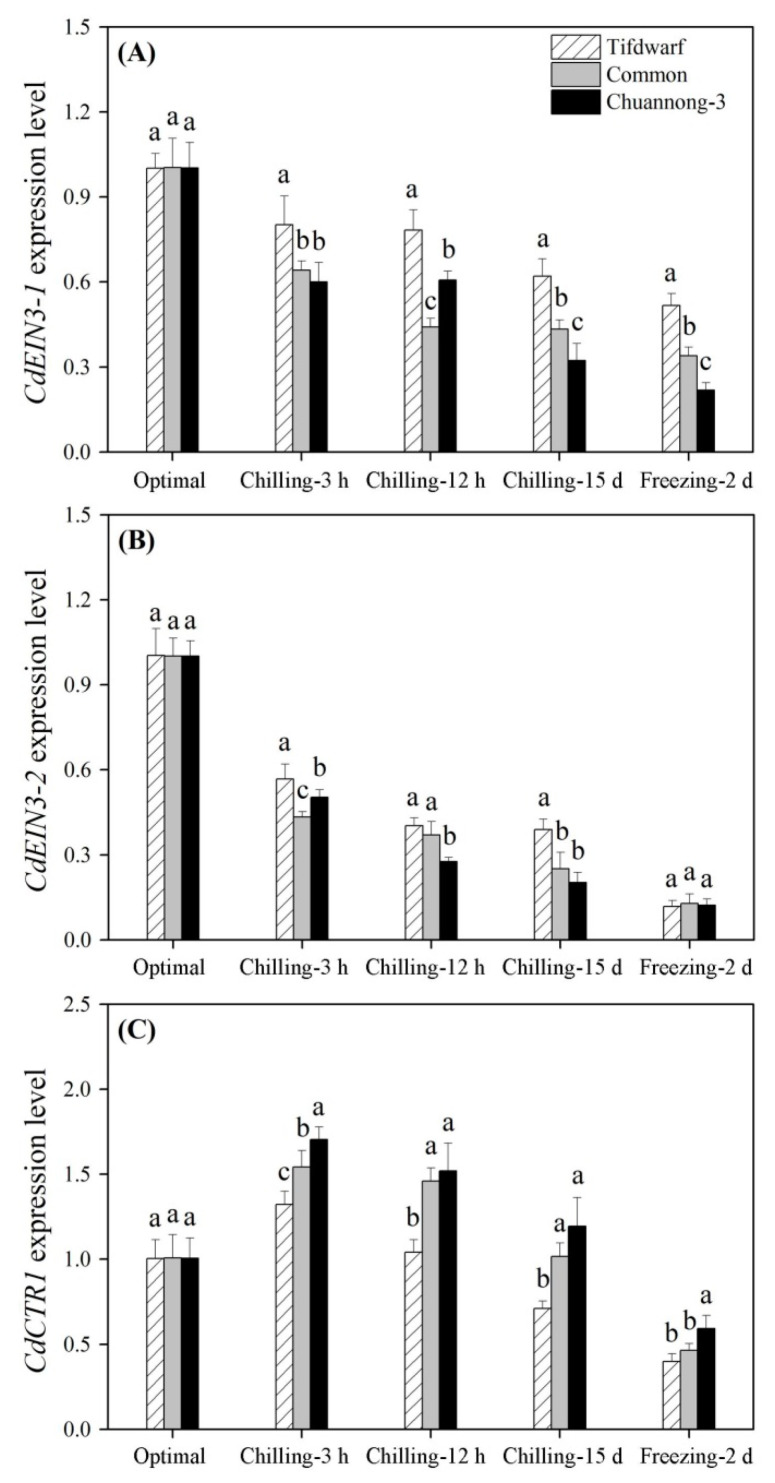
Changes in expression levels of (**A**) *CdEIN3-1*, (**B**) *CdEIN3-2*, and (**C**) *CdCTR1* among three bermudagrass genotypes (Tifdwarf, common bermudagrass, and Chuannong-3) under optimal, chilling, and freezing conditions. Vertical bars indicate positive standard error of mean (*n* = 4). Different letters above columns indicate significant differences under optimal, chilling, or freezing condition based on least significant difference (*p* < 0.05), respectively.

Expression levels of *CdEIN3-1* and *CdEIN3-2* encoding ethylene insensitive proteins gradually declined from 3 h of CS to 2 d of FS (Figure 7A,B). Tifdwarf had a 1.3 times higher *CdEIN3-1* expression level than common bermudagrass or Chuannong-3 at 3 h of CS (Figure 7A). The highest *CdEIN3-1* expression level was also detected in the Tifdwarf at 12 h of CS, on the 15th d of CS, and on the 2nd d of FS (Figure 7A). Tifdwarf exhibited a 1.6 times higher *CdEIN3-2* expression level than common bermudagrass or Chuannong-3 on the 15th d of CS, but there was no significant difference in *CdEIN3-2* expression levels under FS (Figure 7B). The *constitutive triple response 1* (*CdCTR1*) expression level in the three genotypes reached its the maximum at 3 h of CS and then decreased gradually to the minimum on the 2nd d of FS (Figure 7C). The highest *CdCTR1* expression level was observed in Chuannong-3 during CS and FS (Figure 7C).

**Figure 8:**
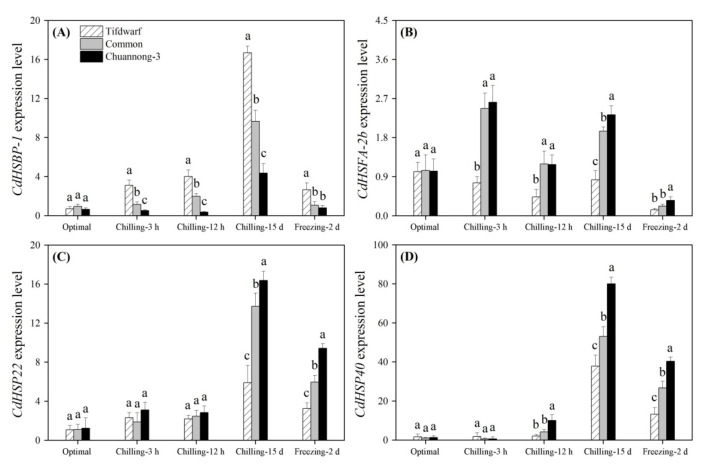
Changes in expression levels of (**A**) *CdHSBP-1*, (**B**) *CdHSFA-2b*, (**C**) *CdHSP22*, and (**D**) *CdHSP40* among three bermudagrass genotypes (Tifdwarf, common bermudagrass, and Chuannong-3) under optimal, chilling, and freezing conditions. Vertical bars indicate positive standard error of mean (*n* = 4). Different letters above columns indicate significant differences under optimal, chilling, or freezing condition based on least significant difference (*p* < 0.05), respectively.

**Figure 9:**
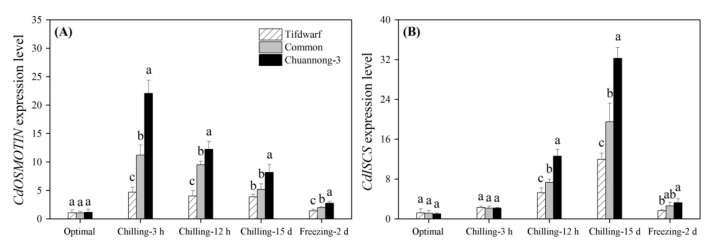
Changes in expression levels of (**A**) *CdOSMOTIN*, and (**B**) *CdISCS* among three bermudagrass genotypes (Tifdwarf, common bermudagrass, and Chuannong-3) under optimal, chilling, and freezing conditions. Vertical bars indicate positive standard error of mean (*n* = 4). Different letters above columns indicate significant differences under optimal, chilling, or freezing condition based on least significant difference (*p* < 0.05), respectively.

**Figure 10:**
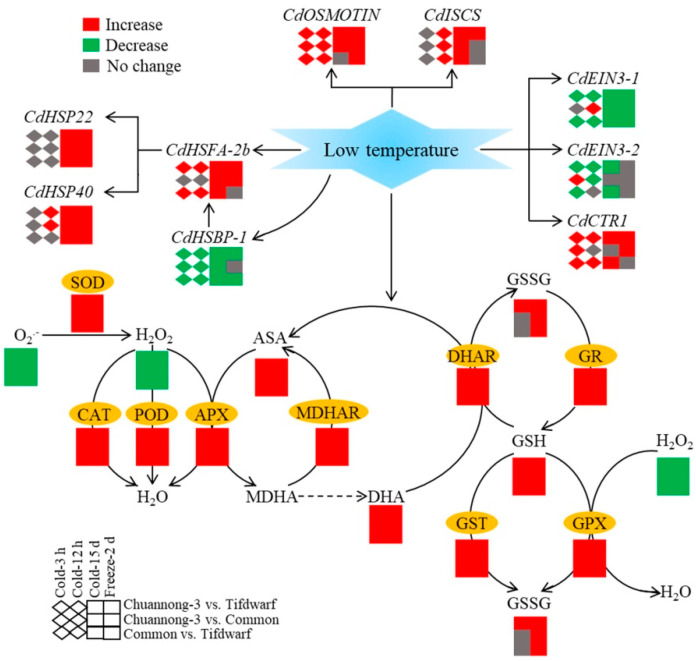
Integrative pathways involved in antioxidant metabolism and cold-responsive genes in three bermudagrass genotypes (Tifdwarf, common bermudagrass, and Chuannong-3) under chilling and freezing conditions.

CS (3 h and 12 h) significantly induced *heat shock factor binding protein 1* (*CdHSBP-1*) expression in Tifdwarf and common bermudagrass but inhibited its expression in Chuannong-3 (Figure 8A). Tifdwarf had a 1.7 and 3.8 times higher *CdHSBP-1* expression than common bermudagrass and Chuannong-3 on the 15th d of CS, respectively (Figure 8A). In addition, the highest *CdHSBP-1* expression level was also detected in the Tifdwarf rather than the other genotypes under FS (Figure 8A). Common bermudagrass and Chuannong-3 showed significantly higher *heat shock factor A-2b* (*CdHSFA-2b*) expression level compared to Tifdwarf during CS (Figure 8B). Chuannong-3, common bermudagrass, and Tifdwarf exhibited the highest, the second highest, and the lowest expression levels of *CdHSP22* and *CdHSP40* under CS (15 d) and FS (2 d), respectively (Figure 8C,D). CS significantly up-regulated the *CdOSMOTIN* expression level in all genotypes, and the amount of increase was highest in Chuannong-3 (Figure 9A). The expression level of the *CdISCS* encoding a cysteine desulfurase gradually increased from 3 h to 15 d under CS and then declined under FS in the three genotypes (Figure 9B). The peak value of *CdISCS* expression level was detected in Chuannong-3 on the 15th d of CS (Figure 9B). Figure 10 demonstrates the integrative pathways involved in antioxidant metabolism and cold-responsive genes in three bermudagrass genotypes under CS and FS conditions.

## 3. Discussion

Cold stress triggers various pathways in the plants. Among them, the activation of the antioxidant defense system is one of the most important adaptive strategies for plants to prevent damage from cold-induced overaccumulation of ROS [31,32,33]. A positive correlation between enhanced antioxidant defense and cold tolerance in bermudagrass has been reported. The study of Zhang et al. stated that antioxidant enzyme activities were positively correlated with the freezing tolerance of two common bermudagrass cultivars: Riviera and Princess-77 [34]. Similar responses were observed in wild bermudagrass which up-regulated SOD and POD activities as well as the transcript levels of genes encoding these antioxidant enzymes in relation to the mitigation of CS damage [35]. The exogenous application of plant growth regulators including melatonin, nitric oxide, and ABA effectively improved the cold tolerance of bermudagrass by enhancing the antioxidant defense systems [8,36,37,38]. Our current study found that CS and FS increased the accumulation of MDA and also decreased cell membrane stability in three bermudagrass genotypes as a result of the oxidative damage induced by overproduction of O_2_^−^ and H_2_O_2_. Three bermudagrass genotypes primarily increased SOD, POD, APX, and GST activities to alleviate cold-induced oxidative damage. As two critical nonenzymatic antioxidants in plants, ASA and GSH are reduced to DHA and GSSG by the APX and GST, respectively, resulting in H_2_O_2_ scavenging in cells [10]. MDHAR regulated the regeneration of ASA, and the overexpression of a *BrMDHAR* improved the freezing tolerance of *Arabidopsis thaliana*, which was associated with maintenances of high ASA content and activities of APX, DHAR, GR, and GPX under FS [39]. As compared to Tifdwarf and common burmudagrass, Chuannong-3 possessed a more effective ROS scavenging system to establish a better redox homeostasis when subjected to CS and FS. In addition, ROS accelerated the degradation of Chl, thereby reducing photochemical efficiency. More stable photosynthetic pigments and delayed leaf senescence were positively related to lower oxidative damage in chickpea plants under CS [13]. The strong antioxidant defense system effectively depressed the accumulation of ROS in Chuannong-3 in favor of the mitigation of cold-induced adverse effects on Chl and photochemical efficiency.

Gaseous signal molecules, including ETH and H_2_S, transduce cold signaling to regulate antioxidant metabolism, sugar accumulation, the CBF pathway, etc., thereby impacting tolerance to CS and FS. However, ETH mainly acts as a negative regulator of cold tolerance while H_2_S plays a positive role in plant adaptation to cold stress [19,40,41]. Exogenous application of an ETH precursor reduced the chilling tolerance of wild-type bermudagrass, as reflected by the reduced photosynthetic performance and antioxidant capacity. On the contrary, the ETH antagonist Ag^+^ alleviated chilling-induced oxidative damage and Chl loss [22]. The study of Shi et al. also proved the negative effects of ETH on tolerance to FS in *Arabidopsis thaliana* by repressing the cold-induced CBF1 pathway [21]. *CTR1* encoding a constitutive triple response 1 which is a Raf-family-like protein kinase negatively regulates ETH signaling, but ethylene insensitive 3 (EIN3) is a positive regulator in the ETH pathway [42,43,44]. In response to CS and FS, a continued slowdown in transcript levels of *CdEIN3-1* and *CdEIN3-2* was detected in three bermudagrass genotypes; however, CS induced a significant increase in *CdCTR1* expression in leaves of Tifdwarf, common burmudagrass, and Chuanong-3 in the current study. The *Arabidopsis thaliana ein3-1* mutant avoided ETH-induced leaf senescence [42]. *EIN3* overexpression weakened the freezing tolerance of *Arabidopsis thaliana* by downregulating *CBF1* expression, but the *ein3-1* mutant showed enhanced freezing tolerance [21]. ETH activated expressions of *EIN3-1* and *EIN3-2* along with downregulating *CBF1* in bermudagrass under CS [22]. It is noteworthy that cold-tolerant Chuannong-3 and common bermudagrass maintained significantly lower *CdEIN3-1* and *CdEIN3-2* expressions, as well as higher *CdCTR1* expression, than cold-sensitive Tifdwarf during CS and FS. These findings, together with previous studies, indicate the negative role of the ETH signaling pathway in the mediation of CS and FS tolerances in bermudagrass. Similarly, ETH signaling activated EIN3 to transcriptionally repress the CBF1 pathway when soybean (*Glycine max*) was subjected to CS [40].

H_2_S has been identified as a defensive signaling molecule conferring cold tolerance in various plant species [41]. For example, a H_2_S scavenger attenuated the adaptability of *Vitis vinifera* seedlings to CS [24]. Overexpression or knockdown of a key *cysteine desulfhydrase* gene involved in H_2_S biosynthesis increased or decreased endogenous H_2_S content in *Arabidopsis thaliana*, followed by an improved or reduced chilling tolerance, respectively [25]. H_2_S activated the expression of chilling-responsive genes such as *CBF3*, *COR15A*, and *COR15B* in favor of chilling tolerance in *Arabidopsis thaliana* [45]. Similarly, exogenous ammonia borane effectively alleviated CS damage to rapeseed (*Brassica napus*) seedlings through the H_2_S-regulated antioxidant system and CBF-COR pathway [46]. H_2_S-indcued increases in ROS-scavenging enzyme activities including, SOD, CAT, and APX, were propitious to mitigate ROS injury to hawthorn (*Crataegus monogyna*) fruit under CS [47]. *ISCS*, encoding a cysteine desulfurase, is responsible for endogenous H_2_S biosynthesis [48]. However, limited studies have been conducted on the function of *ISCS* against CS and FS through the H_2_S-mediated mitigation of ROS or other pathways in bermudagrass and other species. In the current study, CS up-regulated the expression of *CdISCS* in three bermudagrass genotypes, and the increase was the highest and lowest in cold-tolerant Chuannong-3 and the cold-susceptive Tifdwarf genotype, respectively. This indicated that *CdISCS* could be involved in cold-induced H_2_S signaling for the defensive response to CS in bermudagrass, but it is still worth further investigating the potential role of *CdISCS* in regulating chilling or freezing tolerance in our future study.

It has been reported that the detrimental effect of ETH on the chilling tolerance of common bermudagrass could be associated with a decreased abundance of HSPs in leaves [49]. One of the important functions of HSPs is to improve the correct refolding of denatured proteins and to prevent the aggregation of impaired proteins. HSFs are transcriptional activators which control *HSPs’* expression in favor of plant tolerance to fluctuating ambient temperature [50]. HSBPs interacting with HSFs attenuate HSF’s DNA-binding capacity, thus depressing *HSPs* expression [51,52]. The chilling tolerance of cucumber (*Cucumis sativus*) plants could be significantly enhanced through the overexpression of *CsHSF1d* which activated the ICE–CBF–COR pathway, whereas *CsHSFA1d* knockdown lines exhibited sensitive phonotypes under CS [53]. Multiple *HSFs* and *HSPs* were up-regulated by CS in common bermudagrass, indicating a possible positive role of the heat shock pathway in chilling tolerance [54]. Similar results were found in the current study, which demonstrated that the best cold tolerance of Chuannong-3 was accompanied by the highest transcript levels of *CdHSFA-2b*, *CdHSP22*, and *CdHSP40*, as well as the lowest *CdHSBP-1* expression in comparison to other genotypes after being subjected to cold stress. In addition, a previous study by Wang et al. also found that FS induced expressions of a large number of *HSFs* and possible HSF-regulated target genes such as *HSPs*, *APXs*, and *galactinol synthase* (*GOLS1*) involved in raffinose biosynthesis in tall fescue (*Festuca arundinacea*) and perennial ryegrass (*Lolium perenne*) [55]. The overexpression of *ZmHSFA2* significantly mitigated the deleterious effects of high temperature associated with the upregulation of many genes related to raffinose biosynthesis for the accumulation of raffinose in maize (*Zea mays*). In this process, ZmHSBP2 acted as an antagonist of ZmHSF2 [56]. Our previous study has also shown that the enhanced cold tolerance of Chuannong-3 was related to the improvement in raffinose biosynthesis for osmotic adjustment and osmoprotection [30].

Osmotin and osmotin-like proteins belonging to the pathogenesis-related type-5 protein family not only protect plants against pathogens, but also play a major role in abiotic stress tolerance due to their beneficial functions of osmoregulation, molecular chaperone for the repair of denatured proteins, and mediation of stress-defensive genes as a transcriptional regulator [57]. *Osmotin* has been identified and cloned from different plant species and its positive role in cold tolerance has also been studied widely. For instance, the cryoprotective function of *osmotin* in olive trees (*Olea europaea*) was related to chilling acclimation [58]. ABA upregulated the expression of an osmotin-like gene, *pAl3*, for the achievement of chilling tolerance in *Solanum commersonii* [59]. Transgenic tomato (*Solanum lycopersicum*) overexpressing an *osmotin* cloned from tobacco (*Nicotiana tabacum*) improved the transcript abundance of *CBF1* and *APX*, thus contributing to better tolerance than the wild type after exposure to CS [60,61]. The constitutive expression of a *Tripogon loliiformis osmotin* (*TlOsm*) improved tobacco seedling establishment and survival rate under CS [62]. The current findings show that CS quickly induced *CdOSMOTIN* expression in three bermudagrass genotypes. The best, intermediate, and worst cold tolerance of Chuanong-3, common bermudagrass, and Tifdwarf was consistent with the highest, medium, and the lowest expression level of *CdOSMOTIN* in response to cold stress, indicating that the regulation of *CdOSMOTIN* could be an effective strategy for adaptability to CS and FS in bermudagrass species. 

## 4. Materials and Methods

### 4.1. Plant Materials and Treatments

Stems of Tifdwarf, Chuannong-3, and common bermudagrass were collected from a farm at Sichuan Agricultural University and planted in a tube of 20 cm length and 11 cm diameter. All tubes contained some type and amount of loamy soils with 90% particle sizes between 0.002 and 0.02 as well as sands with particle sizes between 0.1 and 0.2 mm (1:1). A total of 15 stems were evenly cultivated in each tube which was placed randomly in the greenhouse for one and a half months of establishment. During this time, the plants in each tube were irrigated by 100 mL of Hoagland’s nutrient solution twice a week and trimmed once or twice a week to maintain a 4 cm mowing height [63]. All plants were then removed into controlled growth chambers (30/25 °C, 750 μmol m^−2^ s^−1^ PAR, and 65% humidity) for 10 days of acclimation. Leaf samples were collected for the control treatment under optimal condition. All plants were subjected to 20/16 °C (day/night) for 2 days, 16/12 °C for another 2 days, and then 8/4 °C for 15 days as the treatment under chilling condition. After that, the temperature in the growth chambers was set at 2/−2 °C for 2 days for the freezing condition treatment. For physiological parameters and enzyme activities, samples were collected under optimal, chilling (15 days), and freezing (2 days) conditions. For genes’ expression levels, samples were collected under optimal, chilling (3 h, 12 h, and 15 days), and freezing (2 days) conditions. Each genotype included four tubes (four replications) which were separated into four growth chambers. Three genotypes were arranged randomly in the growth chamber.

### 4.2. Determination of Chlorophyll Content, Photochemical Efficiency, and Oxidative Damage

A total of 0.2 g of fresh leaves were soaked in 15 mL of dimethyl sulfoxide to extract Chl, and the absorbance of leach liquor was detected at 663 nm and 645 nm by using a spectrophotometer (Spectronic Instruments, Rochester, NY, USA) [64]. Fv/Fm and PI_ABS_ were detected by using a Chl fluorescence meter (Pocket PEA, Hansatech, Norfolk, UK) [65]. O_2_^−^ and H_2_O_2_ content were determined according to the method of Elstner and Heupel [66] and Velikova et al. [67], respectively. For the determination of EL, 0.1 g of fresh leaves were cleaned by deionized water and then soaked in 35 mL of deionized water for 24 h at room temperature (25 °C). The initial conductivity of the solution was detected by using a conductivity meter (YSI Model 32, Yellow Spring, OH, USA). The leaves were killed at 120 °C for 15 min and then the final conductivity of the solution was detected. The EL was calculated based on the ratio of initial conductivity to the final conductivity [68]. The MDA content was detected by using the method of Dhindsa et al. [69]. Briefly, fresh leaves (0.1) were ground with 2 mL of 50 mM phosphate-buffered saline (PBS, pH 7.8) to form homogenates. After being centrifuged at 10,000× *g* for 20 min, the supernatant was mixed with reaction solution, and then the mixture was heated at 100 °C for 15 min. The absorbance of the supernatant was detected at 532 and 600 nm after the mixture was centrifuged at 8000× *g* for 15 min.

### 4.3. Determination of Antioxidant Enzyme Activity and Antioxidant Metabolite

Fresh leaf (0.1 g) was ground in 2 mL of PBS to retrieve homogenate, which was centrifuged at 10,000× *g* for 20 min; then, the supernatants were used for the determination of antioxidant enzyme activity. For the determination of SOD activity, 0.5 mL of the supernatants were mixed with 50 mM PBS containing 195 mM methionine, 60 mM riboflavin, and 1.125 mM NBT, and the absorbance of the mixture was detected at 560 nm [70]. POD and CAT activities were determined by using the method of Chance and Maehly [71]. For APX activity, 0.5 mL of supernatant was mixed with 1.5 mL of PBS (pH 5.8) solution containing 10 mM ascorbic acid, 0.003 mM EDTA, 5 mM H_2_O_2_, and 100 mM. The absorbance of mixture was recorded every 10 s for 1 min at 290 nm [72]. MDHAR, DHAR, and GR activities were detected using the method of Cakmak et al. [73]. GST activity, GPX activity, GSH content, GSSG content, ASA content, and DHA content were detected by using assay kits which were purchased from Grace Biotechnology (Suzhou, China) according to manufacturer’s instructions. The protein content of enzyme extracts was analyzed by using Bradford’s method [74].

### 4.4. Determination of Gene Expression

A total of 0.1 g of fresh leaf was collected from each replication of each treatment and then the total RNA was extracted by using an RNeasy Mini Kit. These RNAs were then reverse-transcribed to cDNA for gene amplification in a qRT-PCR detection system (iCycler iQ qRT-PCR detection system with SYBR Green Supermix, Bio-Rad, Hercules, CA, USA) at the following settings: 1 min at 95 °C and 39 repeats of denaturation at 95 °C for 20 s, annealing at 55–62 °C (Table S1) for 20 s, following by heating the amplicon from 60 to 95 °C to obtain the melting curve. The amplification system included SYBR Green Supermix, primers, and hyperpure water. The primer sequences and annealing temperature of *CdEIN3-1*, *CdEIN3-2*, *CdCTR1*, *CdHSFA-2b*, *CdHSBP-1*, *CdHSP22*, *CdHSP40*, *CdOSMOTIN*, *CdISCS*, and *CdACTIN2* as a reference gene were recorded in Table S1. The calculation of gene expression levels was based on formula 2^−ΔΔCt^ [75]. Three biological and three technical replicates were used for each gene.

### 4.5. Statistical Analysis

Data were recorded and organized using Excel 2019 (Microsoft Office 2019, Albuquerque, NM, USA). Differences among treatment means were tested using Fisher’s protected least significance (LSD) test at a 0.05 probability level [30].

## 5. Conclusions

The three bermudagrass genotypes exhibited significant variation in chilling and freezing tolerances based on the evaluations of Chl content and photochemical efficiency in PS II. Chuannong-3 and Tifdwarf showed the best and worst cold tolerance, and the cold tolerance of common bermudagrass 001 was ranked as an intermediate between Chuannong-3 and Tifdwarf. Chuannong-3 achieved better tolerance through enhancing the antioxidant defense system to stabilize cell membranes and ROS homeostasis after being subjected to CS and FS. Chuannong-3 also down-regulated the ETH signaling pathway by improving *CdCTR1* expression and suppressing the transcript levels of *CdEIN3-1* and *CdEIN3-2*, but up-regulated the H_2_S signaling pathway via an increase in *CdISCS* expression under CS and FS. In addition, the molecular basis of cold tolerance of Chuannong-3 could be associated with the mediation of key genes in the heat shock pathway (*CdHSFA-2b*, *CdHSBP-1*, *CdHSP22*, and *CdHSP40*) and *CdOSMOTIN*. The current findings provide valuable insight into the physiological and molecular mechanisms of cold tolerance in the new cultivar Chuannong-3.

## Data Availability

Not applicable.

## Supplementary Materials

The following supporting information can be downloaded at: https://www.mdpi.com/article/10.3390/ijms241814070/s1.
