# Supplementary material for: Differential Regulations of Antioxidant Metabolism and Cold-Responsive Genes in Three Bermudagrass Genotypes under Chilling and Freezing Stress
Source: Int J Mol Sci. 2023 Sep 14;24(18):14070. doi: 10.3390/ijms241814070 (PMC10530996; doi:10.3390/ijms241814070)
Supplement: Supplementary file 1 [file ijms-24-14070-s001.zip › ijms-2484399-supplementary.pdf]

Table S1 Primer sequences and annealing temperature of genes.

Target gene	Forward primer (5'-3')	Reverse primer (5'-3')	Tm (℃)
CdEIN3-1	GCAGCACTGCGACCCGCCGCA	ACTTGGACTGCCGGACCAGGC	62
CdEIN3-2	GTGGTTGCGTCTTCAGTTGTT	GCACTTGAAGAGCTGACCATA	56
CdCTR1	AGAAAGCTGTTGCTGGTGACG	CTGCAGCAAGTGATAGATCAC	57
CdHSFA-2b	TGAGATGAGAAAGGAGCTCCAAGAGGCC	CAACAAAACAAAATCAGTTGCTGCCTAAAGGT	62
CdHSBP-1	ACCTCAAGGCTGAAATGG	GCCACCAGCAACTGTAAT	55
CdHSP22	AACACGGGAAAGCAAACG	AGGAGGAGCAGCGCAAGGA	56
CdHSP40	TAATAGGTGCAGGAAGCC	TTTCACAGGGAACAAACC	56
CdOSMOTIN	GTGCCATTGTCCTTCTCTTG	CGCAGGGATGGTTCTTAGAG	56
CdISCS	ACGTCATGAAGCTGCAGATC	CCATTTGTTTTCCCTTCACCC	56
CdACTIN2	TCTGAAGGGTAAGTAGAGTAGTAG	ACTCAGCACATTCCAGCAGAT	55
